# Disturbance, neutral theory, and patterns of beta diversity in soil communities

**DOI:** 10.1002/ece3.1313

**Published:** 2014-12-02

**Authors:** Stefanie Maaß, Massimo Migliorini, Matthias C Rillig, Tancredi Caruso

**Affiliations:** 1 Institut für Biologie, Plant Ecology, Freie Universität BerlinAltensteinstraße 6, 14195, Berlin, Germany; 2 Berlin-Brandenburg Institute of Advanced Biodiversity Research (BBIB)14195, Berlin, Germany; 3 Department of Life Sciences, University of Sienavia Aldo Moro 2, Siena, 53100, Italy; 4 School of Biological Sciences, Queen's University of Belfast97 Lisburn Road, Belfast, BT9 7BL, Northern Ireland

**Keywords:** Beta diversity, disturbance, microarthropods, neutral models, oribatid mites, soil, spatial and temporal change

## Abstract

Beta diversity describes how local communities within an area or region differ in species composition/abundance. There have been attempts to use changes in beta diversity as a biotic indicator of disturbance, but lack of theory and methodological caveats have hampered progress. We here propose that the neutral theory of biodiversity plus the definition of beta diversity as the total variance of a community matrix provide a suitable, novel, starting point for ecological applications. Observed levels of beta diversity (BD) can be compared to neutral predictions with three possible outcomes: Observed BD equals neutral prediction or is larger (divergence) or smaller (convergence) than the neutral prediction. Disturbance might lead to either divergence or convergence, depending on type and strength. We here apply these ideas to datasets collected on oribatid mites (a key, very diverse soil taxon) under several regimes of disturbances. When disturbance is expected to increase the heterogeneity of soil spatial properties or the sampling strategy encompassed a range of diverging environmental conditions, we observed diverging assemblages. On the contrary, we observed patterns consistent with neutrality when disturbance could determine homogenization of soil properties in space or the sampling strategy encompassed fairly homogeneous areas. With our method, spatial and temporal changes in beta diversity can be directly and easily monitored to detect significant changes in community dynamics, although the method itself cannot inform on underlying mechanisms. However, human-driven disturbances and the spatial scales at which they operate are usually known. In this case, our approach allows the formulation of testable predictions in terms of expected changes in beta diversity, thereby offering a promising monitoring tool.

## Introduction

Ecological communities are not homogenous in space and time for a number of reasons: dispersal processes, stochastic demographic fluctuations, environmental filtering, niche partitioning processes, and biotic interactions within and between trophic levels interact to determine variable patterns of covariation in species distribution (Hubbell 2001; Chase and Leibold 2003; Morin 2011; HilleRisLambers et al. 2012). Disturbance is one of the processes that contribute to the spatial and temporal heterogeneity of communities (Walker 2012): If communities are equilibrium assemblages of coexisting species (Chase and Leibold 2003; Morin 2011), disturbance prevents assemblages from reaching the equilibrium state. This process can create a long-lasting state of nonequilibrium conditions that promote diversity (e.g., the intermediate disturbance hypothesis; Connell 1978). Communities can also be governed by processes such as chaotic dynamics (May 1973; Morin 2011) where populations are regulated by deterministic factors but are very sensitive to initial conditions: Even the smallest change in the initial state leads to strongly diverging temporal trajectories of population densities. In this case, disturbance can affect initial conditions (e.g., the initial abundance of certain species) by continually resetting them, thereby contributing to rendering the assembly process highly uncertain and variable in terms of the species that locally come together to form assemblages. Communities could also be assembled purely through stochastic processes such as those assumed in neutral theories (Bell 2001; Hubbell 2001). In this latter set of theories, processes such as niche partitioning are just ignored when predicting basic community properties such as variation in species richness or species spatial turnover (Condit et al. 2002). In this case, disturbance can take the form of, for example, increased habitat fragmentation, which is expected to reduce dispersal, thereby increasing beta diversity.

Recently, ecologists have become interested in the effects of disturbance on the spatial distribution of coexisting species. Metacommunity frameworks (Leibold et al. 2004) are useful as they consider a set of local communities embedded in a landscape and connected by dispersal processes within a matrix that might experience heterogeneous conditions, for example, but not only, in terms of environmental gradients. In this framework, local communities are assembled under different forces, and different assembly processes (species sorting, mass effects, and patch dynamics) can be described depending on the relative effects of these forces, which interact as follows: The environment locally filters dispersing species, which might interact with each other under niche partitioning processes but can also be supported by immigration if dispersal rates are adequate. Disturbance might alter these processes either via affecting dispersal (e.g., isolation of patches via habitat fragmentation) or via increasing spatial heterogeneity in environmental conditions, or both. These two effects of disturbance can take place at different scales, as in the case of soil communities (Ettema and Wardle 2002): In soil, local communities can be defined at very fine scales such as the rhizosphere of a single plant. Also, steep gradients in variables such as pH, oxygen, and nutrients are observable already over a few centimeters (Bardgett 2005). At larger scales, such as those relevant to fire or agricultural practices such as tillage, disturbance can either increase or decrease environmental heterogeneity. For example, in the case of high-temperature fire, the intensity of disturbance is patchily distributed, increasing environmental heterogeneity within the extent of the fire. On the other hand, agriculture is a homogenizing disturbance that mixes up soil vertically while it horizontally reduces the diversity of organic input, patchiness, or gradients in the distribution of nutrients: For example, the establishment of monocultures represents an homogenizing environmental factor at a landscape scale (Wardle 2002; Bardgett 2005; Walker 2012).

If spatial heterogeneity determines heterogeneity in the composition of local assemblages, we also expect disturbance to increase beta diversity if it also increases environmental heterogeneity (Dornelas et al. 2006; Caruso et al. 2012a,b). For the same principle and on the other hand, if disturbance homogenizes spatial properties, we expect a decrease in beta diversity. In this sense, very different mechanisms such as a neutral assembly processes versus niche assembly processes (as well as other processes discussed above) can lead to the same pattern given the factor causing the pattern (disturbance). This offers high potential for applications such as environmental monitoring and conservation (Anderson et al. 2006, 2011; Dornelas et al. 2006; Caruso et al. 2012a,b) because disturbance is expected to cause recurrent patterns in beta diversity, regardless of the mechanisms governing the assembly process. Thus, the simplest set of processes (e.g., neutral dynamics) can offer a baseline to detect disturbance, as we argue below.

However, two problems potentially hamper applications: First, beta diversity has proved to be a multifaceted and even controversial concept (Legendre et al. 2005; Tuomisto and Ruokolainen 2006; Anderson et al. 2011; Legendre and De Cáceres 2013); second, testing for differences in beta diversity is complicated by the numerous ways in which beta diversity can be measured, and the statistical and dynamical (sensu community dynamics) links between beta diversity and gamma diversity (Kraft et al. 2011; Legendre and De Cáceres 2013; Myers et al. 2013).

**Figure 1:**
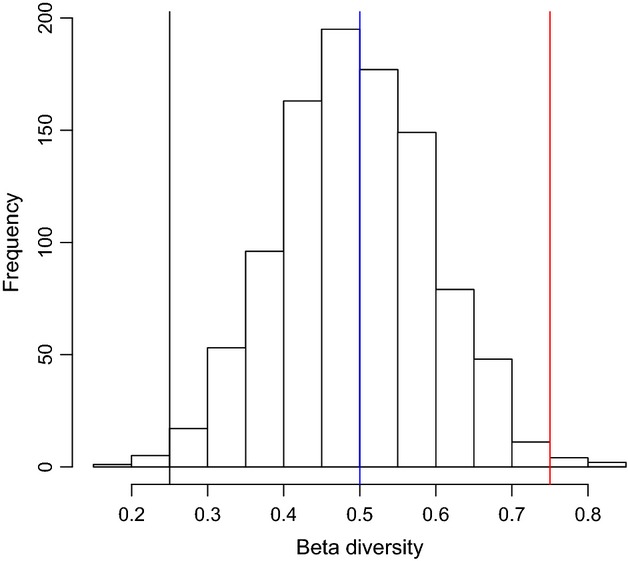
This conceptual figure shows the qualitative idea behind the method applied in this study: The beta diversity of a real set of local assemblages (lines) can be similar to (blue line), higher than (red line), or smaller than (black line) the mean of a distribution of beta diversity values obtained from a neutral model. Neutral models assume simple population dynamics that provide background levels of beta diversity, with a mean and a variance. However, real dynamics, based on processes such as environmental filtering, can make real communities significantly diverge (red line) or converge (black line) relative to their neutral counterpart.

We here propose two solutions, based on some of the ideas that have been recently discussed in the field: (1) We apply the recent definition of beta diversity as total variance of the community matrix (Legendre and De Cáceres 2013); (2) we use a general neutral model to create a null prediction of beta diversity under the simplest metacommunity scenario (Dornelas et al. 2006; Etienne 2007; Gotelli and Ulrich 2012). There are several advantages to this approach. Beta diversity is summarized in one number that is easy to calculate and interpret. Most importantly, this number is not computed from alpha and gamma diversity while its statistical dependency on gamma and alpha diversity (Kraft et al. 2011) is taken into account through the use of a general neutral model. We here use such a model to produce a statistical null distribution of beta diversity based on fundamentals of population dynamics (Rosindell et al. 2012). Observed beta diversity can be compared to this distribution (Fig. 1).

Here we test this approach on our own datasets that describe soil oribatid mites under several disturbance regimes and a range of natural, undisturbed environments. Oribatid mites together with collembolans represent the most diverse and abundant group of soil microarthropods: These mites play a key role in soil organic matter decomposition (Scheu 2002; Bardgett 2005; Maraun et al. 2011; Caruso et al. 2012b) and have been studied extensively with regard to both testing general assembly models in soil assemblages (Anderson 1975; Lindo and Winchester 2009; Nielsen et al. 2010; Caruso et al. 2012b) and investigating the response of soil animals to human activities (Behan-Pelletier 1999; Caruso et al. 2008; Al-Assiuty et al. 2014). Soil assemblages possess interesting metacommunity properties: They are assembled at multiple spatial scales, and several species have limited dispersal capability (Ettema and Wardle 2002). For this reason, the assembly of taxa such as oribatid mites has been studied in the framework of the debate around neutral theories (Lindo and Winchester 2009; Caruso et al. 2012b). We here quantify the beta diversity of oribatid mite assemblages under several types and regimes of disturbance and natural environmental heterogeneity. Given mechanisms that were already known to be likely to operate, these different disturbances or conditions were expected to produce variable levels of beta diversity that, depending on disturbance type and/or environmental conditions, could be lower than, higher than, or consistent with beta diversity levels predicted by a general neutral model. We here also provide our original R scripts and relevant data to show how to apply the method, and we discuss how results may inform about ecological applications, in particular the monitoring of communities subjected to disturbance regimes.

## Material and Methods

Our original aim was to use results from a literature search using the Web of Science and the following key words: oribatid*, abundance, distribution* pattern*, soil, community, structure* (in various combinations). We wanted to include all studies on European oribatid soil fauna in nonextreme habitats since 1950. Unfortunately, after this search, at least as to August 2013, only very few studies reported the species abundance table that is necessary to fit neutral models, and very often, these few studies reported data for a low number of replicates. We therefore decided to base our analysis on our own datasets, one of which is unpublished while the others were the subject, to different extents, of previous publications (Migliorini et al. 2002; Caruso et al. 2005, 2009; Caruso and Migliorini 2006). Eventually we were able to compile twelve datasets: Six of them were obtained from undisturbed areas (a beech forest, two grasslands, the thin, rocky, undifferentiated soils of two arid Mediterranean islands, the control plot of a Mediterranean maquis subjected to experimental fires), the other six datasets were obtained from metal-polluted soils, experimentally burned plots, coppice, a badland and heathland resulting from agriculture activities. In the case of metal-polluted soils, the pollution gradient was very steep already at small scales (Caruso et al. 2009), and we could expect diverging assemblages in this case. Even moderate fires usually cause very patchy disturbance regimes, due to the irregular distribution of fire intensity (Caruso and Migliorini 2006), and therefore, we expected diverging assemblages also in this case, and both within and between plots. In the case of land management, we could expect either converging assemblages (i.e., homogenized assemblages) given the scale at which we sampled or diverging assemblages depending on the land use.

The species abundance distribution of each sample (i.e., the local community) was used to estimate the two main parameters of neutral theory: theta (*θ*), an index of diversity, and immigration rate (I). We used the formula for multiple samples by (Etienne 2007) to estimate neutral parameters using the PARI/GP codes given in Etienne (2007). With the estimated parameters, we used the Pari/GP function urn2.gp (Etienne 2007) to create 4999 neutral equivalents of each dataset, which eventually allowed us to create a null distribution of beta diversity for each dataset. For the estimate of neutral parameters and the function urn2.gp, see Data S1. The output of this analysis is the input for the R script reported in Data S2. Beta diversity (BD) was quantified using the approaches proposed by Legendre and De Cáceres (2013). These authors propose to quantify beta diversity as the sum of species variances in the species by site matrix (see Data S1 for a full definition), the latter matrix being the typical outcome of community studies. As this definition of beta diversity implies that the ecological dissimilarity between sites is Euclidean, data must be properly transformed to be ecologically meaningful. Alternatively, meaningful ecological distance matrices can be computed from the raw data and used to estimate BD. This is the most important, central aspect and advantage of this definition of BD, which makes beta diversity a quantitative measure capable of capturing the variation described in the past through a multitude of often redundant dissimilarity indices (see also table 1 in Legendre and De Cáceres (2013). The metric proposed by Legendre and De Cáceres (2013) seems particularly useful because it fits well into two main aspects of neutral models: Spatial changes in species composition are due to dispersal processes, and the variance in species abundance is caused by stochastic demographic fluctuations.

**Table 1:** Characteristics of the twelve assemblages tested. Bold effect sizes were significant at *P* ≤ 0.05 (see Fig. 2)

Study^1^	*N*^2^	Habitat	Spatial scale	Beta diversity factors	Effect size^3^
S1a	10	Beech forest stand	20 × 20 m plot	Natural, undisturbed area	1.04
S1b	10	Grass stand	20 × 20 m plot	Natural, undisturbed area	0.89
S1c	10	Coppice stand	20 × 20 m plot	Disturbed by cutting	**1.70**
S1d	10	Heathland	20 × 20 m plot	Heterogenous	**1.99**
S1e	10	Badland	20 × 20 m plot	Homogeneous, dry	0.92
S2	36	Dry Grassland	15 × 15 m plot	Natural, undisturbed	−0.79
S3a	22	Lampedusa Is., rocky soil	20 km^2^	Very heterogeneous	**2.49**
S3b	10	Linosa Is., rocky soil	150 m transect	Elevation gradient	**4.36**
S4	24	Grass stand	10 × 40 m plot	Metal pollution	−0.42
S5a	9	Mediterranean Maquis	Three 10 × 5 m plots	Control experimental fire	**1.51**
S5b	9	Mediterranean Maquis	Three 10 × 5 m plots	High-intensity fire	**2.25**
S5c	9	Mediterranean Maquis	Three 10 × 5 m plots	Low-intensity Fire	0.15

1 References for major details on the study areas and methods: S1, Migliorini et al. 2002; S2, unpublished, see methods; S3, Caruso et al. 2005; S4, Caruso et al. 2009; S5, Caruso and Migliorini 2006.

2 *N* is the number of local communities (independent soil samples).

**Figure 2:**
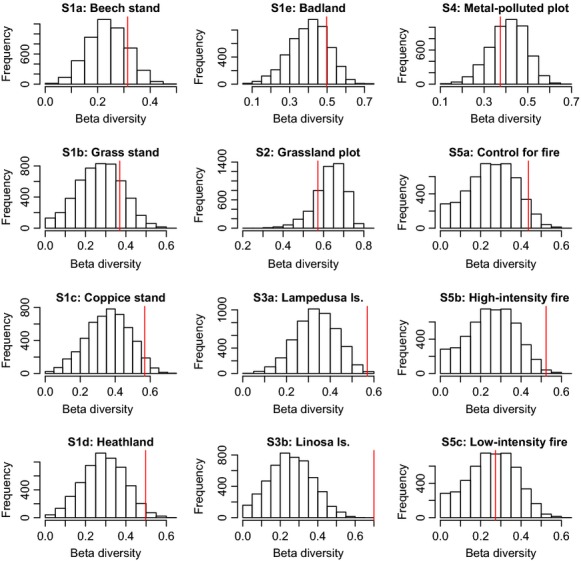
The observed beta diversity (red line) is compared to the frequency distribution of 4999 beta diversity values obtained from simulated neutral communities. Beta diversity is computed as the total community variance of the Hellinger transformed species by sites abundance table (Legendre and De Cáceres 2013; Data S1). The parameters used to simulate neutral communities were estimated from the real data using Etienne (2007).

3 Effect size was equal to [BD-Mean (simulated BDs)]/standard deviation (simulated BDs), BD being beta diversity and simulated BDs being the distribution of BDs obtained for each of the 4999 simulated neutral communities (Fig. 2).

There are many options for both data transformation and distance matrices (Legendre and De Cáceres 2013). We here apply the Hellinger transformation, which has several advantages (Legendre and De Cáceres 2013): (1) The relevant Euclidean distance matrix can be analyzed by principal component analysis (PCA) or canonical redundancy analysis (RDA); (2) the calculation of BD on raw data after transformation is straightforward; (3) BD ranges from 0 to 1; and (4) Hellinger transformation allows to calculate the “species contribution to beta diversity” statistics. Additionally, the Hellinger transformation does not inflate the weights of rare species (Legendre and Gallagher 2001).

BD was calculated using the R function provided in Legendre and De Cáceres (2013). This statistic was calculated on the real datasets, and each of the 4999 neutral datasets simulated for each dataset. If the observed BD was higher or lower than 95% of the simulated datasets, the observed (real) community was considered to have respectively higher or lower beta diversity than expected under neutral assembly (Fig. 1). Otherwise, data were consistent with neutral dynamics. Given the regime of disturbance or degree of environmental heterogeneity was known for each dataset, results could be interpreted in terms of expected dynamics and outcomes.

## Results

Six of twelve datasets had beta diversity (BD) significantly higher (Fig. 2, see red line) than the null distribution obtained by calculating BD on the 4999 datasets generated with the neutral model of Etienne (2007). These datasets were S1c (Coppice), S1d (Heathland), S3a and S3b (Lampedusa and Linosa), S5a (Control Fire Experiment), and S5b (High-intensity Fire). The effect sizes reported in Table 1 were in these cases significant at *P* ≤ 0.05, meaning that less than 5% of simulated BDs were larger than the observed BD. In the other six cases, the effect size was not significant: In two of these cases, the Dry Grassland and the Metal polluted plots observed BD was smaller than the mean of simulated BDs, while in the remaining four cases, observed BD was larger.

## Discussion

Disturbance generally is detrimental to soil biodiversity (Walker 2012; Ponge and Salmon 2013), especially in agroecosystems, where it is usually intense and frequent. In fact, the spatial homogenization caused by activities such as tillage reduces biological diversity in space and time: A few species eventually dominate the system. On the other hand, natural soils are highly heterogeneous at multiple spatial scales (Ettema and Wardle 2002) and already over a few centimeters (Wardle 2002; Bardgett 2005), and certain regimes of disturbance can actually increase spatial heterogeneity (Walker 2012). Accordingly, several soil taxa are characterized by high species turnover and variance in abundance, that is to say high beta diversity (Lindo and Winchester 2009; Caruso et al. 2012b; Ponge and Salmon 2013). Here we show that six of the twelve tested oribatid mite assemblages diverge relative to the reference point provided by a general neutral model. If we assume that background levels of beta diversity depend on the basic processes postulated by neutral theories (dispersal and stochastic demographic fluctuations), our result means that real communities have higher beta diversity than expected under neutral dynamics. Note that this fact does not imply that communities consistent with neutrality have low beta diversity.

In the other six cases, beta diversity was consistent with neutral predictions. When neutral models are used to build a null distribution and data do not reject the null hypothesis (Rosindell et al. 2012), nothing certain can actually be said on underlying mechanisms (Gotelli and Ulrich 2012). Communities could be neutrally assembled, but possible issues of statistical power or inadequate sampling strategy could also be invoked to explain the results.

Whatever the actual mechanism, the main point of our results is that there is a clear qualitative, simple explanation of why certain assemblages diverge relative to neutral predictions: When disturbance is expected to increase the heterogeneity of soil spatial properties or the sampling strategy encompassed a range of diverging environmental conditions, we observed diverging assemblages. On the contrary, we observed patterns consistent with neutrality when disturbance could determine homogenization of soil properties in space or the sampling strategy encompassed fairly homogeneous areas. Etienne (2007) suggested that one of the reasons why currently available general neutral models might fail in terms of predicting beta diversity is that these models are spatially implicit, even when they allow estimating single dispersal rates for each local assemblage. Disturbance and/or environmental heterogeneity can therefore contribute to the failure of neutral models via affecting assemblages selectively in space, with closer localities that are subject to similar disturbance intensity and frequency. In this sense, it is interesting to pinpoint specific results from disturbed or undisturbed areas that were consistent with neutral expectations. The metal-polluted plot and low-intensity fire, for example, were consistent with neutral predictions. The metal-polluted plot (Caruso et al. 2009) was 40 × 10 m, and within this area, basic soil parameters (e.g., water content, pH, C) and vegetation were fairly homogenous. Metals such as Pb, Zn, and Cu did show steep gradients over 40 m, but we had previously shown that these gradients did not correlate with oribatid mite distribution after removing spatial autocorrelation (Caruso et al. 2009). The collected local assemblages can therefore be seen as random variation around the same core assemblage, which might explain the consistency between neutral predictions of beta diversity and observed beta diversity. The same applies to the data obtained from a 15 × 15 m plot in a dry grassland plot. In theory, one direction of the plot was aligned with an environmental gradient, and assemblages might therefore be expected to diverge. In practice, assemblages did not diverge significantly relative to a neutral model, and we hypothesize that this depends on the small scale of the sampling, not sufficient to encompass the environmental divergence that could make local assemblages significantly diverge. On the other hand, we could have observed convergence: The environment is fairly homogeneous, and the assemblage should therefore converge to the equilibrium expected for the given environmental condition. The observed negative effect size (Table 1) indicated some degree of convergence, but the difference was not statistically significant. Issue of statistical power may apply to this case. The same issue possibly applies to data collected in natural beech and grass stands which were not disturbed: Also in these two cases, the spatial scale of the sampling was relatively small although larger than that of the dry grassland plot. In this case, the effect size indicated some degree of divergence, but again results were not significant (Table 1).

We can therefore understand the nonrejection of neutral models in terms of either the sampling scale of the study and/or statistical power. This seems reinforced by the cases where we did observe significant divergences: In coppice and heathland that were sampled at the same scale as the beech and grass stands, we did observe significant divergence, which is consistent with the high heterogeneity associated with the tree harvests and the management of heathland. The divergence observed in the extremely dry, thin, and rocky soil of Lampedusa (different habitat types sampled within the island; see Caruso et al. 2005) and Linosa (a transect along an elevational gradient) islands can be interpreted in a similar way: In this case, the sampling strategy aimed at maximizing environmental gradients and heterogeneity.

An interesting set of comparisons is that of the three assemblages from the fire experiments: The control assemblages show beta diversity higher than the neutral prediction. The low-intensity fire communities were consistent with the neutral model. The high-intensity fire resulted in beta diversity much higher than the neutral prediction (compare the three effect sizes in Table 1 and Fig. 2). Relative to low fire intensity, high-intensity fire produced a very patchy disturbance with patches that were much more intensely burned than other patches (personal observation): We attribute the observed differences to this effect.

Overall, the results support the general hypothesis that neutral models allow detecting changes in beta diversity caused by disturbance regimes that increase environmental heterogeneity or by natural environmental heterogeneity, which is usually captured at broad scales (>100 m; e.g., Lampedusa and Linosa, Table 1).

There are technical aspects relevant to our interpretation of results and possible applications that are avenue for future research. Neutral models provide a robust null hypothesis because they can provide estimates of beta diversity based on the simplest metacommunity scenario. However, neutral models can be used to detect disturbances in two different ways: First, data reject neutral predictions because the real assemblages vary too much or too little in terms of species composition and abundance (Dornelas et al. 2006; Caruso et al. 2012a,b); second, communities are really assembled under neutral dynamics, and disturbance directly affects neutral parameters, for example, by decreasing dispersal via increasing habitat fragmentation (Hubbell 2001) or by affecting some fundamental demographic parameters (Dornelas 2010). In this study, we basically used neutral models in the first sense because we believe that in observational studies, robust conclusions can be obtained only when sound statistical null hypotheses are rejected (Gotelli and Ulrich 2012). We also believe that in the framework of observational studies, our modeling approach does not allow identifying mechanisms but rather monitoring changes given expectations that come from background knowledge on the study area.

We use a quantitative definition of beta diversity, but one can further simplify the concept by focusing just on compositional aspects, which is done using indices such as the Jaccard index. In this case, community variance (Legendre and De Cáceres 2013) would reflect just changes in species composition across the study area.

In this sense, an interesting aspect to be investigated is the partitioning of beta diversity in terms of pure compositional variation and pure variance in species abundance, a topic that, as far as we are aware, has been introduced in the seminal paper by Anderson et al. (2006) but never analyzed in terms of applications. Ecologically, these two aspects can imply fairly diverse scenarios. Local assemblages can be very different in terms of species composition even if the spatial variance of each species is low and vice versa. In theory, a set of local assemblages can have zero BD if the assemblages are identical in terms of species composition and BD is measured using presence/absence data. However, species abundance usually has some associated variance, and if BD is measured using metrics that take into account quantitative information, BD will not be zero. A key aspect of many definitions of disturbance is that disturbance implies some change in the biomass or abundance of the disturbed population (Walker 2012). This suggests that, especially for applications relevant to the monitoring of the effects of human-induced disturbance, a quantitative approach to BD is worth using to increase our ability to detect effects and eventually interpret them. Theoretical studies based on simulations and accompanied by relevant field experiments are the tools to validate this method in the future. In the meantime, we propose to monitor the effect of disturbance on community structure and the effect of restoration practices using the following seven steps procedure: (1) Assess whether the disturbance regime under investigation increases or decreases environmental heterogeneity and/or environmental predictability and fragmentation; (2) sample local communities at the range of scales pertinent to the disturbance regime; (3) estimate beta diversity using the metrics proposed by Legendre and De Cáceres (2013); (4) fit a general neutral model to species abundance data in order to create a null prediction of beta diversity (Etienne 2007; Data S1); (5) use the null distribution to assess whether the sampled assemblages are diverging or converging relative to their theoretical neutral counterpart, or the assemblages could be consistent with the neutral model (Data S2); (6) assess: if the assemblages are diverging under a disturbance regime that increases heterogeneity or converging under a disturbance regime that homogenizes the environment, then the conclusion is that disturbance is deeply affecting community dynamics with effects on species abundance and composition; (7) plan of action: arrange for replicating observations of the disturbed community in time, also in connection with restoration regimes. If beta diversity is quantified using BD by Legendre and De Cáceres (2013) on Hellinger transformed data, species most responsible for changes in beta diversity can be identified.

## Data Accessibility

Fitting neutral model: key information and references uploaded as Supporting Information. Examples of Species by sample matrices used to generate Fig. 2: uploaded as Supporting Information. R script to estimate beta diversity and create the null distribution for the test proposed in the paper: uploaded as Supporting Information. Example data files and relevant meta data to apply the proposed analysis: uploaded as Supporting Information. Faunistic data (e.g., species names) and details on the soil animal datasets here used to apply the proposed method: data archived in Migliorini et al. (2002); Caruso et al. (2005); Caruso and Migliorini (2006); Caruso et al. (2009).

## Supporting Information

**Data S1.** Supplementary methods to fit neutral models and test predictions of Beta diversity (BD).

**Data S2.** r scripts to calculate BD and perform a null model test based on neutral model predictions.

**Data S3.** Observed species by sample matrix of Heathland (Figure 2, S1d). See Data S1.

**Data S4.** Simulated neutral species by sample matrices for Heathland. See Data S1.
