# Supplementary material for: Disturbance, neutral theory, and patterns of beta diversity in soil communities
Source: Ecol Evol. 2014 Dec 2;4(24):4766–74. doi: 10.1002/ece3.1313 (PMC4278825; doi:10.1002/ece3.1313)
Supplement: Supplementary file 1 — Data S1. Supplementary methods to fit neutral models and test predictions of Beta diversity (BD). [file ece30004-4766-sd1.docx]

**Supporting On-line Material**

Fitting Neutral Model

We used the method and Pari/Gp functions provided by Etiene (2007) to estimate the two parameters (neutral diversity *θ* and immigration *m*) of the general neutral model (Hubbell 2011) applied to the species abundance distribution observed in the field. Etienne (2007) provided a sampling formula for multiple samples and the data we provide for each study area are the species by sample matrices to which this formula applies. The example given here is based on the data set of the Heathland (Figure 2, S1d) and the species by sample matrix of this data set is given in the supplementary file “observed.txt” (Data S4). In this matrix, rows are samples/sites and columns are species. In order to estimate *θ* and *m* with the Pari/Gp functions of Etienne (2007), such a matrix needs to be converted into a string following the example given in Etienne (2007). However, once the parameters were estimated, we simulated neutral communities using the function urn2.gp (Etienne 2007) with the following two lines of code:

read("urn2.gp")

for(n=1,4999,urn2(1.92,[1.72,2.00,9.11,1.01,2.56,2.14,1.69,2.46,5.12,1.23],[154,118,86,110,41,108,106,42,19,27],1))

The output of this is given in the supplementary file simData.txt. Note that a new run of these lines will give a different output because of the newly simulated assemblages. However, the average behaviour of properties such as beta diversity is not affected if the number of simulated communities is reasonably high. The urn2.gp string given above is run via a loop that simulates communities 4999 (in practice the function is applied 4999 times). The function takes three parameters: the estimate of *θ* (in this case 1.92), the estimate of *m* for each sample/site (in this case the vector [1.72,2.00,9.11,1.01,2.56,2.14,1.69,2.46,5.12,1.23], and the total number of individuals per sample (in this case the vector [154,118,86,110,41,108,106,42,19,27]). Note that actually the immigration parameter is not given as in Hubbell (2001), that is to say in terms of immigration rate, but instead it is given in terms of number of immigrants, called *I* by Etienne (2007). The relationship between *m* and *I i*s

$$m=\frac{I}{I+J-1}$$

To summarise, at the end of the neutral model fitting exercise, two files are necessary to complete the analysis proposed in this paper with the R script provided in Supporting Information 2: observed.txt (the species by sample matrix obtained from the field; Data S4) and simData.txt (the simulated communities based on the estimate of neutral parameters; Data S5).
